# Editorial: Methods and applications in emotion regulation and processing

**DOI:** 10.3389/fnbeh.2023.1215119

**Published:** 2023-05-16

**Authors:** Ana Cicvaric

**Affiliations:** Dominick P. Purpura Department of Neuroscience, Albert Einstein College of Medicine, New York, NY, United States

**Keywords:** emotion regulation (ER), emotional states of stress, machine learning, fear conditioning, intracranial electrical self-stimulation, mindfulness-pain intervention

The field of emotion research has experienced significant growth in recent years. Nevertheless, there is no conclusive consensus on what emotion is (LeDoux, 2012). Moreover, while some experts suggest that there is a limited list of basic emotions, there is a lack of agreement on which emotions should be considered as such. Neuroscience studies the influence of emotions on judgment, decision-making, and memory, as well as trying to understand the reasons and outcomes of uncontrolled emotional reactions, which deviate from the usual range of emotional responses and affective disorders (Ortony, 2022).

This Research Topic on “*Methods and applications in emotion regulation and processing*” includes four articles covering a wide range of methods utilized to study emotion-related behaviors in both rodents and humans. The neuroscience toolbox is constantly expanding, which enables us to ask and answer increasingly more complex questions. This Research Topic provides a valuable guide on how to take full advantage of recent advancements to achieve an unbiased, standardized approach in both data collection and analysis in neuroscience, specifically when studying complex behaviors.

Vila-Solés et al. show that intracranial electrical stimulation of the medial forebrain bundle (MFB) -whether self- and researcher-administered- facilitated learning in the Morris Water maze task. The study also revealed that intracranial electrical self-stimulation (ICCS) and experimenter-administered (EAS) produced different patterns of neuronal activation 72 h post-stimulation. The authors also provide a detailed, standardized step-by-step protocol of intracranial electrical self-stimulation (ICCS) in rats with in-house build equipment. These findings suggest that MFB could be a valuable target for deep brain stimulation in treating memory impairment in humans and reinforce the notion that ICCS in animal models has potential for transitional research.

In their manuscript, Amorim et al. have developed a new self-calibration software, Phobos, which automatizes the measurement of freezing behavior that is commonly used to assess associative fear memory. While many commercially available software packages exist for measuring freezing, they require the user to adjust many parameters leading to variable interresearcher results and increased workload, most of the time at a high cost. Freezing can be easily and reliably quantified by a trained researcher, however, this is a labor-intensive process that does not exclude parameters such as subjectivity and interobserver variability. Phobos offers a solution by leveraging the reliability of manual scoring to produce a calibration video from a single 2 min video that is manually scored by the researcher. The parameters extracted from the calibration file are then used to run batch analysis. This software is freely available, thus contributing to increasing the efficiency and accuracy of scientific research.

Xi et al. show that a combination of empowering education and mindfulness meditation training significantly improved quality of life, medication and lifestyle compliance, and ameliorated negative emotions like anxiety compared to conventional nursing methods in patients with inflammatory bowel disease (IBD). While IBD is primarily a chronic inflammation of the gastrointestinal tract, for which the exact cause is not known, not only have anxiety and depression been frequently reported concomitantly with IBD by the patients, but recent animal and human studies have shown that physiological stressors can trigger flare-ups of symptoms. This study highlights the importance of conducting research that aims at treatment methods in which patients benefit from the synergistic effect, in this case, mindfulness as a core psychological skill for mental health and wellbeing with patients having a more active role in their treatment through empowering education methods.

In the Mini review, Kuo et al. provide a helpful guide for researchers interested in using deep learning techniques to study complex behaviors in rodent models. The review highlights the main characteristics, advantages, and limitations of supervised, unsupervised, and self-supervised machine learning approaches, and provides examples of the most commonly used algorithms in neuroscience. The authors emphasize the importance of machine learning in reducing subjectivity and promoting standardization in scoring emotion-related behaviors, making it a powerful tool that integrated with neural imaging could provide a more nuanced approach to understanding the relationship between emotional states, behavior, and neural activity.

This selection aims to highlight the newest experimental techniques and methods in Emotion Regulation and Processing research, while also providing an easy guide to utilizing cutting-edge technologies. This Research Topic emphasizes the crucial importance of promoting data quality and reproducibility in neuroscience, as well as the importance of dissemination of open-source tools as a means for promoting diversity and making neuroscience more equitable.

## Author contributions

The author confirms being the sole contributor of this work and has approved it for publication.
